# Supplementary material for: Advancing aerogel recyclability through polyhexahydrotriazine reactivity
Source: Nat Commun. 2025 Dec 5;17:371. doi: 10.1038/s41467-025-67059-y (PMC12796227; doi:10.1038/s41467-025-67059-y)
Supplement: Supplementary file 1 — Supplementary Information [file 41467_2025_67059_MOESM1_ESM.pdf]

Supporting Information

Advancing Aerogel Recyclability through Polyhexahydrotriazine Reactivity

Chang-Lin Wang[1], Yi-Ru Chen[1], Brahim Mezari[2], Fabian Eisenreich[1], Željko Tomović[1]*

[1]Polymer Performance Materials Group, Department of Chemical Engineering and Chemistry and Institute for Complex Molecular Systems (ICMS), Eindhoven University of Technology, Eindhoven, The Netherlands.

[2] Inorganic Materials and Catalysis Group, Department of Chemical Engineering and Chemistry, Eindhoven University of Technology, Eindhoven, The Netherlands.

*z.tomovic@tue.nl

Table of Contents

General information 3

Supercritical CO_2 drying setup 3

Synthesis of *N,N*-bis(4-aminobenzyl)terephthalamide (BAPTPA) (i) 4

Synthesis of hexa(acetamidophenyl)cyclophosphazene (HAAPP) (ii) 5

Synthesis of hexa(aminophenyl)cyclotriphosphazene (HAPP) (iii) 5

Synthesis of 1,3,5-tris(4-methoxyphenyl)-1,3,5-triazinane (OMeHT) (iv) 6

Synthesis of 1,3,5-tris(4-ethoxyphenyl)-1,3,5-triazinane (OEtHT) (v) 6

Thermal conductivity analysis of PHT aerogels 7

Supplementary tables 8

Results and Discussion 14

Study of decomposition of PHT-pristine using BAPP solution 14

Investigation of bond exchange reaction between different hexahydrotriazines 14

Investigation of material properties of PHT aerogels after long-term humidity/heat exposure 15

Supplementary figures 16

References 31

General information

Supercritical CO_2 drying setup

The setup for supercritical CO_2 drying was established based on previously reported studies.[1] Liquid CO_2 grade 2.7 (purity > 99.7%) is used as exchange agent for the supercritical drying process (SCD). The high pressure extraction/drying units “HP-DE200” is utilized as the drying setup. It comprises one autoclave, provided by Eurotechnica, with a maximum working temperature of 100 °C and allowable operation pressure of 220 bar. The autoclave includes a thermowell with a NiCr-Ni thermocouple to measure the internal temperature during the process. Two venting tubes are also attached to the autoclave to extract the covering solvent and depressurization. Apart from the autoclave, the supercritical drying system employed in this work consists of two thermal baths (Selecta, UNITRONIC 200) for heat exchanger 1 and 2, a mechanical pump (provided by Maximator), a check number valve, 5 needle valve and a CO_2 bottle.

Gels are first introduced into the autoclave and covered with the solvent used for gel formation. This was done to avoid premature solvent evaporation that could lead to a higher shrinkage. Then, CO_2 is gradually pressurized up to 100 bar, extracting the solvent from the inside of the wet gel pores. The heat exchanger 2 maintains the autoclave at constant temperature of 60 °C. Once supercritical conditions are achieved, the solvent is extracted from the gels in the autoclave. The supercritical CO_2 enriched with extracted solvent is vented out by releasing the autoclave. During this process, the pressure is maintained above with constant fresh CO_2 input. The venting process takes around 10 to 15 min and the autoclave will be closed to reach further extraction. Three cycles of extraction were further conducted with waiting interval of 30 min each. Finally, when the aerogel pores are completely free of solvent, pressure is slowly released to atmospheric pressure through the metering valve for 45 min.

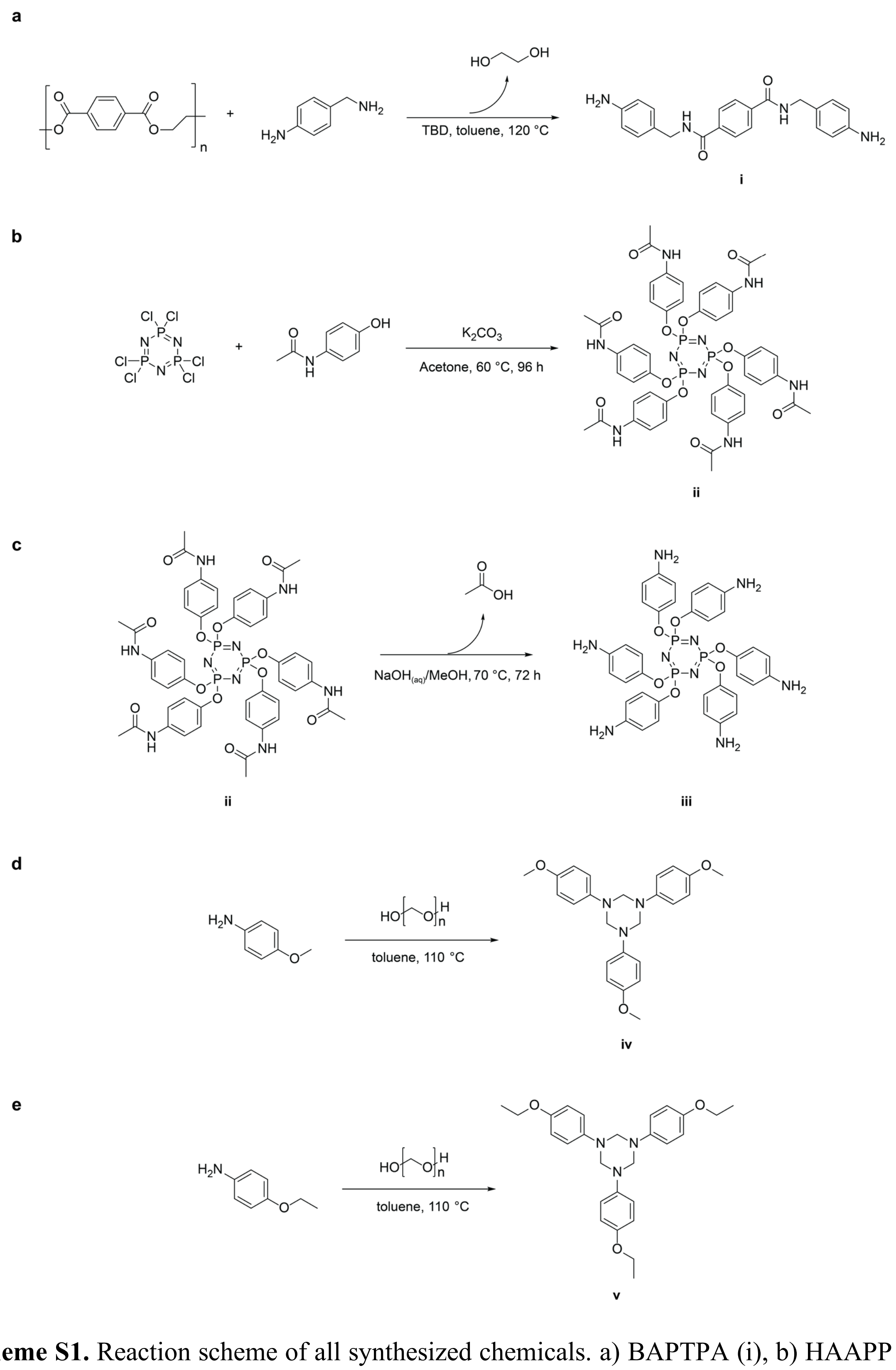

Scheme S1. Reaction scheme of all synthesized chemicals. a) BAPTPA (i), b) HAAPP (ii), c) HAPP (iii), d) OMeHT (iv), e) OEtHT (v).

Synthesis of *N,N*-bis(4-aminobenzyl)terephthalamide (BAPTPA) (i)

PET (20.0 g, 104 mmol, 1 eq.), 4-aminobenzylamine (50.9 g, 416 mmol, 4 eq.) and TBD (1.16 g, 8.3 mmol, 0.02 eq.) were placed in a 100 ml round bottom and then heated under argon atmosphere at 120 °C for 1 h. The mixture was triturated and washed in toluene and isopropanol at 50 °C. The residue was then dried in a vacuum oven at 80 °C, yielding a white powder as a product (28.5 g, 73%).

^{1}H NMR (400 MHz, DMSO-d_6): δ = 4.30 (d, ^{3}J(H,H) = 5.8 Hz, 4H, N-CH_2), 4.94 (s, 4H, NH_2), 6.51 (d, ^{3}J(H,H) = 8.4 Hz, 4H, Ar-H), 6.99 (d, ^{3}J(H,H) = 8.4 Hz, 4H, Ar-H), 7.93 (s, 4H, Ar-H), 8.95 (t, 2H, NH) ppm. ^{13}C NMR (100 MHz, DMSO-d_6): δ = 42.5, 113.7, 126.4, 127.2, 128.4, 147.6, 165.3 ppm. MS (m/z): $[M]^+$ calc. for $C_{22}H_{22}N_4O_2^+$, 374.17; found, 374.18.[2]

Synthesis of hexa(acetamidophenyl)cyclophosphazene (HAAPP) (ii)

In a 2 L three neck round-bottomed flask, 4-acetamidophenol (61.3 g, 0.4 mol, 10.0 eq.) and anhydrous potassium carbonate (70.1 g, 0.5 mol, 12.5 eq.) were dissolved in 1 L of acetone with vigorously stirring, then hexachlorocyclotriphosphazene (13.8 g, 0.04 mol, 1.0 eq.) was added dropwise into solution. The reaction mixture was stirred for an additional 96 hours under reflux. The solid was filtered and the filtrate was collected. After evaporating the solvent, the crude liquid was precipitated in distilled water at 80 °C, and the white powder was dried in vacuum oven at 50 °C overnight. (21.7 g, 0.02 mol, yield 53%).

^{1}H NMR (400 MHz, DMSO-d_6): δ = 2.04 (s, 18H, OC-CH_3), 6.80 (d, ^{3}J(H,H) = 9 Hz, 6H, Ar-OH), 7.44 (d, ^{3}J(H,H) = 9 Hz, 6H, Ar-H), 7.43 (s, 6H, Ar-H), 9.92 (s, 6H, N-H) ppm. ^{13}C NMR (100 MHz, DMSO-d_6): δ = 23.9, 120.1, 120.6, 136.5, 145.1, 168.2 ppm. ^{31}P NMR (161.9 MHz, DMSO-d_6): δ = 9.18 (s, 3P, P=N) ppm. MS (m/z): $[M+H]^+$ calc. for $C_{48}H_{47}N_9O_{12}P_3^+$, 1036.26; found, 1036.24.

Synthesis of hexa(aminophenyl)cyclotriphosphazene (HAPP) (iii)

In a 250 mL three neck round-bottomed flask, HAAPP (13.3 g, 0.01 mol) was dissolved in 200 mL methanol with vigorously stirring, then 40 mL of 10 M NaOH solution was added dropwise into solution. The reaction mixture was stirred for an additional 72 hours under reflux. The solid was filtered and washed with distilled water, and dried in vacuum at 60 °C for 24 h (8.56 g, 0.01 mol, yield 85%).

^{1}H NMR (400 MHz, DMSO-d_6): δ = 4.89 (s, 6H, NH_2), 6.43 (d, ^{3}J(H,H) = 8.8 Hz, 12H, Ar-H), 6.52 (d, ^{3}J(H,H) = 8.8 Hz, 12H, Ar-H) ppm. ^{13}C NMR (100 MHz, DMSO-d_6): δ = 114.2, 120.9, 121.0, 140.8, 145.6 ppm. ^{31}P NMR (161.9 MHz, DMSO-d_6): δ = 10.12 (s, 3P, P=N) ppm. MS (m/z): $[M+H]^+$calc. for $C_{36}H_{37}N_9O_6P_3^+$, 784.20; found, 784.20.

Synthesis of 1,3,5-tris(4-methoxyphenyl)-1,3,5-triazinane (OMeHT) (iv)

p-Anisidine (5.00 g, 40.6 mmol, 1 eq.) and paraformaldehyde (1.34 g, 44.6 mmol, 1.1 eq.) were added to a round-bottom flask and the reagents were dissolved in 0.66 M, 67 mL of toluene. The reaction mixture was stirred at 110 °C under N_2 atmosphere. After 3 h, the solvent was removed under vacuum. The product OMeHT was obtained as a white solid without further purification (5.29 g, 13.1 mmol, 96%).

^{1}H NMR (400 MHz, 25 °C, $CDCl_3$) δ = 7.06–6.96 (m, 2H, Ar-H), 6.83–6.73 (m, 2H, Ar-H), 4.68 (s, 2H, N-CH_2-N), 3.75 (s, ^3J(H,H) = 7.0 Hz, 3H, CH_3) ppm. ^{13}C NMR (100 MHz, 25 °C, $CDCl_3$) δ = 154.61, 142.76, 121.03, 120.49, 120.20, 114.88, 114.57, 114.43, 83.40, 55.65 ppm. MS (m/z): [M−H]$^-$ calc. for $C_{24}H_{26}N_3O_3^-$, 404.20; found, 404.19.

Synthesis of 1,3,5-tris(4-ethoxyphenyl)-1,3,5-triazinane (OEtHT) (v)

p-Phenetidine (5.00 g, 36.4 mmol, 1 eq.) and paraformaldehyde (1.20 g, 40.6 mmol, 1.1 eq.) were added to a round-bottom flask and the reagents were dissolved in 0.66 M, 69 mL of toluene. The reaction mixture was stirred at 110 °C under N_2 atmosphere. After 3 h the reaction was complete and the solvent was removed under vacuum. The product OEtHT was obtained as a brown solid without further purification (5.27 g, 11.8 mmol, 97%).

^{1}H NMR (400 MHz, 25 °C, $CDCl_3$) δ = 7.00 (d, ^3J(H,H) = 8.9 Hz, 2H, Ar-H), 6.77 (d, ^3J(H,H) = 9.0 Hz, 2H, Ar-H), 4.67 (s, 2H, N-CH_2-N), 3.96 (q, ^3J(H,H) = 7.0 Hz, 2H, CH_2), 1.37 (t, ^3J(H,H) = 7.0 Hz, 3H, CH_3) ppm. ^{13}C NMR (100 MHz, 25 °C, $CDCl_3$) δ = 153.93, 142.73, 120.20, 115.32, 71.29, 63.90, 15.04 ppm. MS (m/z): [M−H]$^-$ calc. for $C_{24}H_{26}N_3O_3^-$, 446.24; found, 446.24.

Thermal conductivity analysis of PHT aerogels

The total thermal conductivity (λ_{Total}) of all aerogels was partitioned into gaseous (λ_g) and solid (λ_s) conductivities using Knudsen's model. The gaseous thermal conductivity was calculated using **Equation 1**:

$$\lambda_g = \frac{\lambda_{g,0} \times \Pi/100}{1 + 2\beta \frac{l_d}{\Phi}} \tag{1}$$

Where $\lambda_{g,0}$ is the thermal conductivity of still air at 1 atm and room temperature (0.02619 W $m^{-1}K^{-1}$); Π is the porosity (**Table S4**); β is the gas-solid energy exchange efficiency factor (assumed to be 2); l_d is the mean free path of nitrogen gas molecules at experimental conditions (taken as 70 nm); Φ is the average pore diameter, as shown in **Table S4**. Radiative heat transfer was assumed negligible. The solid thermal conductivity was obtained by subtracting the gaseous component from the total, as shown in **Equation 2**:

$$l_s = l_{Total} - l_g \tag{2}$$

Calculated values of λ_g and λ_s are provided in **Table S5**.

Supplementary tables

Table S1. Recovery yields of the solvent used during PHT-pristine synthesis

Components	Yield [%]
Ethanol	96
Water	93
NMP	62

Table S2. General material properties of PHT-pristine

Name	Bulk density ρ_b [mgcm^{-3}]	Linear shrinkage [%][a)]	Skeletal density ρ_s [gcm^{-3}]	Porosity Π [%][b)]	Specific surface area σ_{BET} [m^{2}g^{-1}][c)]	Total pore volume V_{total} [cm^{3}g^{-1}][d)]	Mesopore volume V_{BJH} [cm^{3}g^{-1}][e)]	Average pore size Φ [nm][f)]	Total thermal conductivity λ_{total} [Wm^{-1}K^{-1}][g)]	Gaseous thermal conductivity λ_s [Wm^{-1}K^{-1}][h)]	Solid thermal conductivity λ_s [Wm^{-1}K^{-1}][i)]
PHT-pristine	145	17	1.22 ± 0.003	88	129	6.08	0.25	188	0.0188 ± 5e-05	0.0093	0.0095

a) Linear shrinkage was calculated based on the diameter change of the sample; b) Porosity was calculated via equation: $\Pi=(1-\rho_b/\rho_s)\times100\%$; c) Calculated based on BET theory. d) Calculated via $V_{Total} = (1/\rho_b) - (1/\rho_s)$ e) Calculated using the BJH method from the desorption branch of the isotherms; f) Calculated via $\Phi = 4 \times V_{Total}/$BET specific surface area, σ. g) Measured with a heat flow meter; h) Calculated using the Knudsen equation (eqn. 4); i) Calculated via $\lambda s = \lambda_{Total} - \lambda_g$, and assuming that the radiative heat transfer was negligible;

Table S3. Formulation of recycled polyhexahydrotriazine aerogels (PHT-As)

	Depolymerization					Re-gelation	
Name	PHT-pristine [g]	PHT-A1 [g]	PHT-A2 [g]	BAPP [g]	NMP [g]	PFA [g]	NMP [g]
PHT-A1	1.08	-	-	1.52	9	0.22	13.5
PHT-A2	-	1.08	-	1.52	9	0.22	13.5
PHT-A3	-	-	1.08	1.52	9	0.22	13.5
PHT-A1[a)]	0.43	-	-	0.61	3.7	0.09	5.3
PHT-A2[a)]	-	0.43	-	0.61	3.7	0.09	5.3
PHT-A3[a)]	-	-	0.43	0.61	3.7	0.09	5.3

a) The samples were prepared for compression testing.

Table S4. General material properties of PHT aerogels

Name	Bulk density ρ_b [mgcm^{-3}]	Linear shrinkage [%][a)]	Skeletal density ρ_s [gcm^{-3}]	Porosity Π [%][b)]	Specific surface area σ_{BET} [m^{2}g^{-1}][c)]	Total pore volume V_{total} [cm^{3}g^{-1}][d)]	Mesopore volume V_{BJH} [cm^{3}g^{-1}][e)]	Average pore size Φ [nm][f)]
PHT-A1	150	16	1.28 ± 0.007	88	135	5.89	0.21	174
PHT-A2	145	18	1.31 ± 0.007	89	123	6.13	0.21	199
PHT-A3	138	15	1.34 ± 0.007	89	133 ± 1.19[g)]	6.50	0.37	195
PHT-B	126	12	1.33 ± 0.008	91	256	7.18	0.61	112
PHR-C	143	18	1.32 ± 0.002	85	212	6.24	0.57	118
PHT-D	196	26	1.35 ± 0.004	84	208	4.36	0.78	84
PHT-F1	145	14	1.22 ± 0.004	88	123	6.08	0.35	198
PHT-F2	143	13	1.27 ± 0.010	89	137	6.21	0.35	181

[a)] Linear shrinkage was calculated based on the diameter change of the sample; [b)] Porosity was calculated via equation: $\Pi=(1-\rho_b/\rho_s)\times100\%$; [c)] Calculated based on BET theory. [d)] Calculated via V_{Total} = $(1/\rho_b)$ - $(1/\rho_s)$ [e)] Calculated using the BJH method from the desorption branch of the isotherms; [f)] Calculated via $\Phi = 4 \times V_{Total}$/BET specific surface area, σ. [g)] The measurements were repeated two additional times, and the average value and standard deviation were determined.

Table S5. Thermal conductivities of PHT aerogels

Name	Total thermal conductivity λ_{total} [Wm^{-1}K^{-1}]	Gaseous thermal conductivity λ_s [Wm^{-1}K^{-1}]	Solid thermal conductivity λ_s [Wm^{-1}K^{-1}]
PHT-pristine	0.0188 ± 5e-05	0.0093	0.0095
PHT-A1	0.0185 ± 4e-05	0.0088	0.0097
PHT-A2	0.0191 ± 7e-05	0.0097	0.0094
PHT-A3	0.0192 ± 1e-03	0.0096	0.0096
PHT-B	0.0159 ± 2e-05	0.0068	0.0091
PHR-C	0.0171 ± 5e-06	0.0066	0.0105
PHT-D	0.0197 ± 3e-05	0.0051	0.0146
PHT-F1	0.0194 ± 1e-04	0.0095	0.0099
PHT-F2	0.0185 ± 5e-05	0.0092	0.0093

[a)] Measured with a heat flow meter; [b)] Calculated using the Knudsen equation (eqn. 4); [c)] Calculated via $\lambda_s = \lambda_{Total} - \lambda g$, and assuming that the radiative heat transfer was negligible.

Table S6. Water uptake and water contact angle of PHT aerogels

Sample	Water uptake [%]	Contact angle [°]
PHT-pristine	2.1	130 ± 4
PHT-A1	0.7	126 ± 3
PHT-A2	1.9	133 ± 3
PHT-A3	1.5	123 ± 7
PHT-B	1.8	116 ± 9
PHT-C	22.8	98 ± 6
PHT-D	8.3	99 ± 4
PHT-F1	1.7	130 ± 2
PHT-F2	0.9	124 ± 12

Table S7. Thermal & mechanical properties of PHT-pristine, PHT-As, PHT-B, PHT-C, and PHT-D

Name	$T_{d5\%}$ [°C][a)]	R_{793} [%][b)]	Compressive modulus [MPa][c)]	Compression strength at 10% deformation ratio [kPa][d)]
PHT-pristine	334.6	16.5	1.28 ± 0.12	456 ± 3
PHT-A1	336.3	14.6	2.01 ± 0.31	626 ± 47
PHT-A2	344.8	15.6	2.59 ± 0.41	545 ± 51
PHT-A3	343.2	14.5	0.95 ± 0.38	443 ± 16
PHT-B	324.6	12.4	1.03 ± 0.23	455 ± 50
PHT-C	338.2	13.5	1.86 ± 0.49	669 ± 63
PHT-D	356.0	39.8	1.93 ± 0.58	773 ± 35

[a)] Decomposition temperatures at 5% weight loss, respectively. [b)] char residue at 793 °C. [c)] Compressive modulus was calculated from the stress-deformation curve obtained using sample of 25 mm diameter and 15 mm height. [d)] Compressive strength at 10% deformation ratio.

Table S8. Formulation of reprogrammed PHT aerogels (PHT-B, PHT-C, and PHT-D)

	Depolymerization					Regelation		
Name	PHT-pristine [g]	FDA [g]	BAPTPA [g]	HAPP [g]	NMP [g]	PFA [g]	NMP [g]	Gelation time [h]
PIA-B	1.09	1.31	-	-	9.3	0.23	13.2	4
PIA-C	1.13	-	1.46	-	9.7	0.24	12.8	2.5
PIA-D	1.36	-	-	1.22	11.6	0.28	10.9	0.5
PIA-B[a)]	0.47	0.57	-	-	4.0	0.10	5.0	4
PIA-C[a)]	0.45	-	0.59	-	4.9	0.10	5.1	2.5
PIA-D[a)]	0.54	-	-	0.49	5.7	0.11	4.3	0.5

[a)] The samples were prepared for compression testing.

Table S9. Formulation of reprocessed PHT aerogels (PHT-Fs)

	Depolymerization				Re-gelation	
Name	PHT-E1 [g]	PHT-F1 [g]	BAPP [g]	NMP [g]	PFA [g]	NMP [g]
PHT-F1	1.08	-	1.52	9	0.22	13.5
PHT-F2	-	1.08	1.52	9	0.22	13.5
PHT-F1[a)]	0.43	-	0.61	3.7	0.09	5.3
PHT-F2[a)]	-	0.43	0.61	3.7	0.09	5.3

[a)] The samples were prepared for compression testing.

Table S10. Mechanical properties PHT-E1 and PHT-E2 obtained from tensile testing

Name	Young's modulus [GPa]	Tensile strength [MPa]	Elongation at break [%]
PHT-E1	2.16 ± 0.03	57 ± 6	2.8 ± 0.3
PHT-E2	2.21 ± 0.07	62 ± 8	3.0 ± 0.4

Table S11. Thermal properties of PHT-pristine, PHT-Es and PHT-Fs

Name	$T_{g,DMA}$[a)] [°C]	$T_{g,DSC}$[b)] [°C]	$T_{d5\%}$[c)] [°C]	R_{793} [%]
PHT-pristine	N.A.	157.8	334.6	16.5
PHT-E1	173.0	154.9	335.5	18.1
PHT-E2	171.1	151.1	335.2	19.0
PHT-F1	N.A.	160.9	353.7	20.4
PHT-F2	N.A.	160.5	333.5	19.4

[a)] Glass transition temperatures obtained from DSC. [b)] glass transition temperatures obtained from DMA. [c)] decomposition temperatures at 5% weight loss, respectively. [d)] char residue at 793 °C.

Table S12. Swelling measurements of PHT-E1 in different solvents at room temperature

Solvents	chloroform	*n*-hexane	acetone	water	ethanol	methanol	ACN	DMF	DMSO
Swelling ratio [%]	228.4	0.1	3.3	0.7	0.1	1.4	1.3	5.8	2.3
Gel content [%]	90.7	99.9	99.4	99.6	99.8	100.0	100.0	100.0	100.0

Table S13. Mechanical properties PHT-F1 and PHT-F2

Name	Compressive modulus [MPa][a)]	Compression strength at 10% deformation ratio [kPa][b)]
PHT-F1	1.33 ± 0.42	652 ± 22
PHT-F2	1.88 ± 0.61	603 ± 95

[a)] Compressive modulus was calculated from the stress-deformation curve obtained using sample of 25 mm diameter and 15 mm height, [b)] Compressive strength at 10% deformation ratio.

Table S14. Mass balance of PHT aerogels

Name	Yield [%][a)]
PHT-pristine	95.1 ± 0.5
PHT-A1	96.3 ± 1.8
PHT-A2	95.0 ± 0.3
PHT-A3	94.0 ± 0.4
PHT-B	94.7 ± 5.3
PHT-C	91.6 ± 1.9
PHT-D	96.0 ± 2.4
PHT-F1	97.9 ± 1.1
PHT-F2	95.1 ± 0.5

[a)] Calculated based on the mass yield of the final aerogels relative to the initial precursors. The calculation was performed using three samples, each with a diameter of 25 mm and a height of 15 mm. The standard deviation was determined from these replicates.

Table S15. Thermal conductivities of PHT-pristine after humidity treatment

Name	Thermal conductivity, λ [$Wm^{-1}K^{-1}$]
PHT-pristine_0 day[a)]	0.0189
PHT-pristine_9 days	0.0187
PHT-pristine_14 days	0.0187

[a)] PHT-pristine_0 day was prepared according to PHT-pristinne synthesis. The sample was placed in climate chamber at 70 °C and RH 70% for periods of 9 and 14 days, respectively, and the thermal conductivity was recorded.

Results and Discussion

Study of decomposition of PHT-pristine using BAPP solution

To investigate the efficiency of depolymerization of PHT-pristine using 14wt% BAPP solution in NMP. PHT-pristine was grinded and kept in the desiccator before use. 0.105 g of aerogel powders were added to the vial. By adding a BAPP solution dissolved in 0.8 g NMP, the PHT-pristine is partially depolymerized. Various equivalent ratios of BAPP, ranging from 0.5 to 2.0 molar ratio compared to the amine content in the aerogel, were added (**Figure S5**). After the addition, the vial was sealed with parafilm and ultrasonicated. The appearance of the solution mixture was recorded with digital camera and presented in **Figure S5**. After 4 h sonication, it could be observed that only sample PHT-iv and PHT-v, with 1.5 and 2 equivalents of added BAPP, exhibits clear dissolution. It suggests that at least 1.5 equivalents of BAPP are required as the minimum amount for the full dissolution of the aerogels.

Investigation of bond exchange reaction between different hexahydrotriazines

To investigate the bond exchange reaction between hexahydrotriaiznes, two different types of hexahydrotriazines, 1,3,5-tris(4-methoxyphenyl)-1,3,5-triazinane (OMeHT) and 1,3,5-tris(4-ethoxyphenyl)-1,3,5-triazinane (OEtHT) were synthesized. To demonstrate this reaction in solid-phase condition, equimolar amount of OMeHT and OEtHT were mixed together. After that, the solid mixture was placed between two aluminum plates, and the plates were hot-pressed under 12 MPa of pressure at 180°C for 0.5 h, followed by 40 MPa of pressure at 180°C for another 0.5 h. After cooling down, the yielding mixture was tested with NMR spectroscopy and MALDI-TOF analysis to identify its chemical composition. According to the NMR spectra from **Figure S3**, the HT characteristic peak at 4.7 ppm remains intact after heat and pressure treatment. In addition, there is no side product presented in the spectra, suggesting no other side reaction or degradation of the HT. On the other hand, MALDI-TOF analysis reveals that there are four HT products originated from different combinations of OMeHT and OEtHT moieties. This indicates that there is a bond exchange reaction between HT structures under heat and pressure.

Investigation of material properties of PHT aerogels after long-term humidity/heat exposure

To evaluate the mechanical stability and thermal insulation performance of the PHT aerogels under long-term heat and humidity exposure, newly prepared PHT-pristine samples were placed in a climate chamber at 70 °C and 70% relative humidity for two weeks. After this aging treatment, both the thermal conductivity and mechanical properties of the samples were assessed. The mechanical performance was examined by uniaxial compression testing, and the resulting stress–strain curves of PHT-pristine before and after climate chamber exposure were nearly identical. This indicates that the aerogels retain their mechanical robustness even after prolonged exposure to elevated temperature and humidity. Furthermore, the thermal conductivity of the PHT-pristine samples remained consistent with the initial values over the aging period, confirming that their thermal insulation performance is also well-preserved under these conditions (**Table S15**).

Supplementary figures

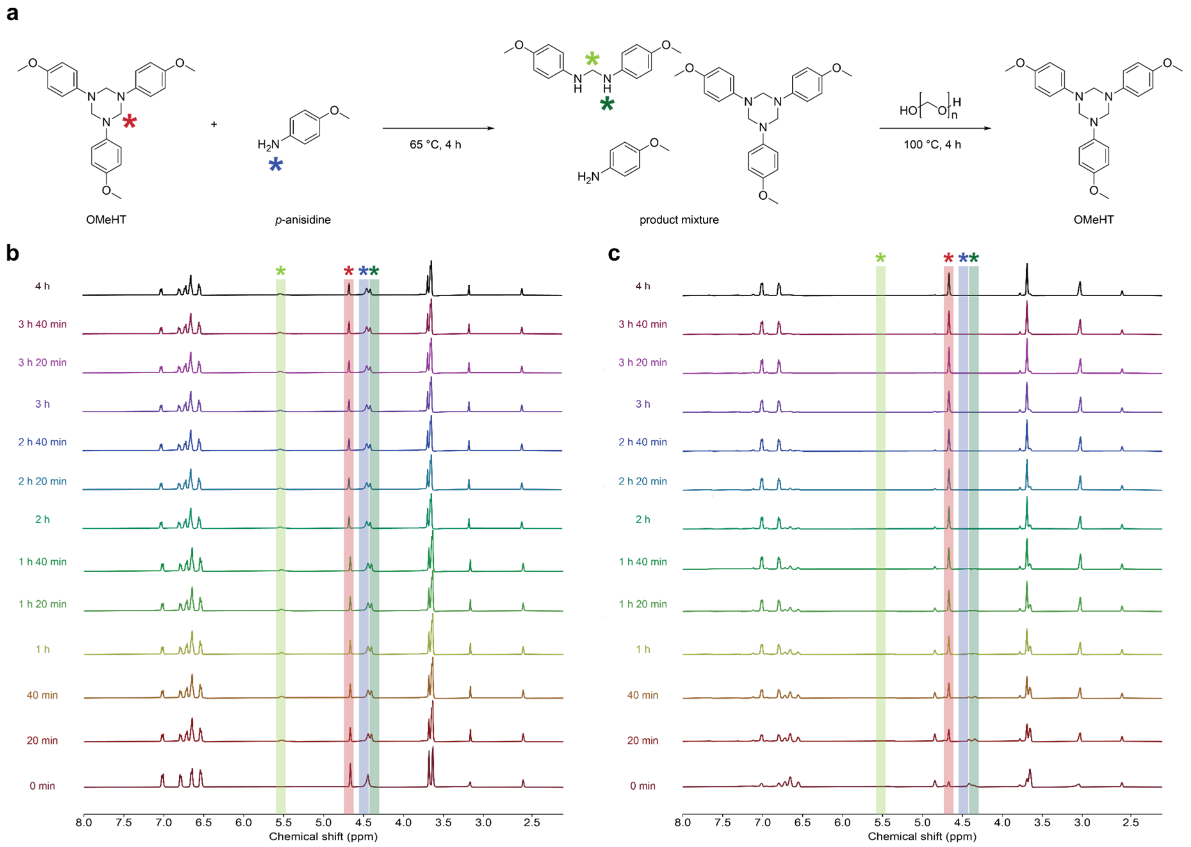

Figure S1. a) Reaction scheme of model reactions between OMeHT, *p*-anisidine and PFA. OMeHT and *p*-anisidine reacted at 65 °C for 4 h in a NMR tube, yielding a product mixture (with possible intermediates). Afterwards, the equivalent amount of repeating units in PFA relative to *p*-anisidine was added to the product mixture and reaction was kept at 100 °C for 4 h. Time-dependent ^{1}H NMR spectroscopy was conducted throughout the model reaction. b) ^{1}H NMR spectra (500 MHz, 65 °C, DMSO-d_6) of the mixture of OMeHT and *p*-anisidine with molar ratio of 1 to 4.5 at 65 °C for 4 h with interval of 20 min. c) ^{1}H NMR spectra (500 MHz, 100 °C, DMSO-d_6) of the mixture of OMeHT and *p*-anisidine after the reaction at 65 °C for 4 h with the addition of an equivalent amount of repeating units in PFA relative to *p*-anisidine at 100 °C for 4 h with interval of 20 min.

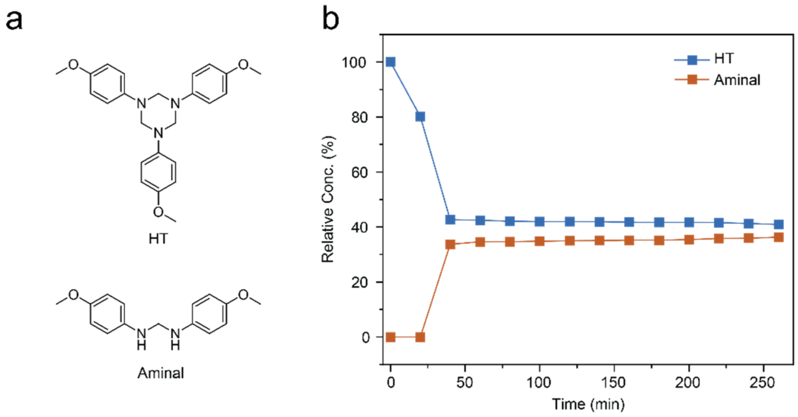

Figure S2. Kinetic profile of the aminolysis reaction between OMeHT and *p*-anisidine. The time-resolved kinetic NMR experiment (500 MHz, 65 °C, DMSO-d_6) was conducted under reaction conditions: OMeHT (1.0 equiv.) and *p*-anisidine (4.5 equiv.) in DMSO-d_6 (40 mM) at 65 °C for 4 h with an acquisition interval of every 20 min.

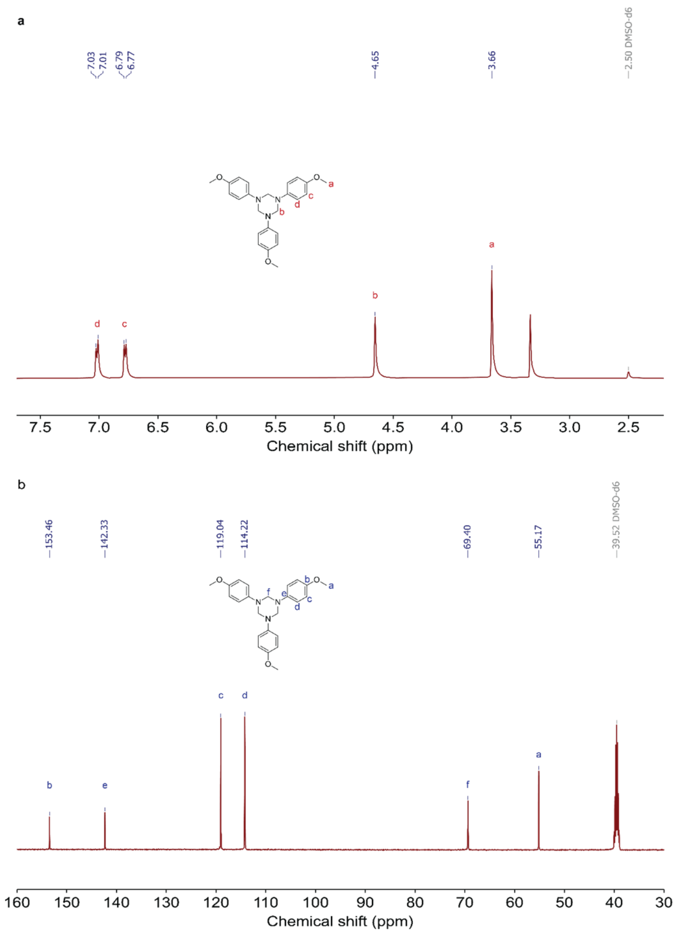

Figure S3. NMR spectra of final product derived from the model reactions between OMeHT, *p*-anisidine and PFA. OMeHT and *p*-anisidine reacted at 65 °C for 4 h in a NMR tube. Afterwards, equimolar amount of PFA relative to *p*-anisidine was added to the product mixture and reaction was kept at 100 °C for 4 h. a) ^{1}H NMR spectra of the final product (500 MHz, 25 °C, DMSO-d_6) b) ^{13}C NMR spectra of the final product (125 MHz, 25 °C, DMSO-d_6).

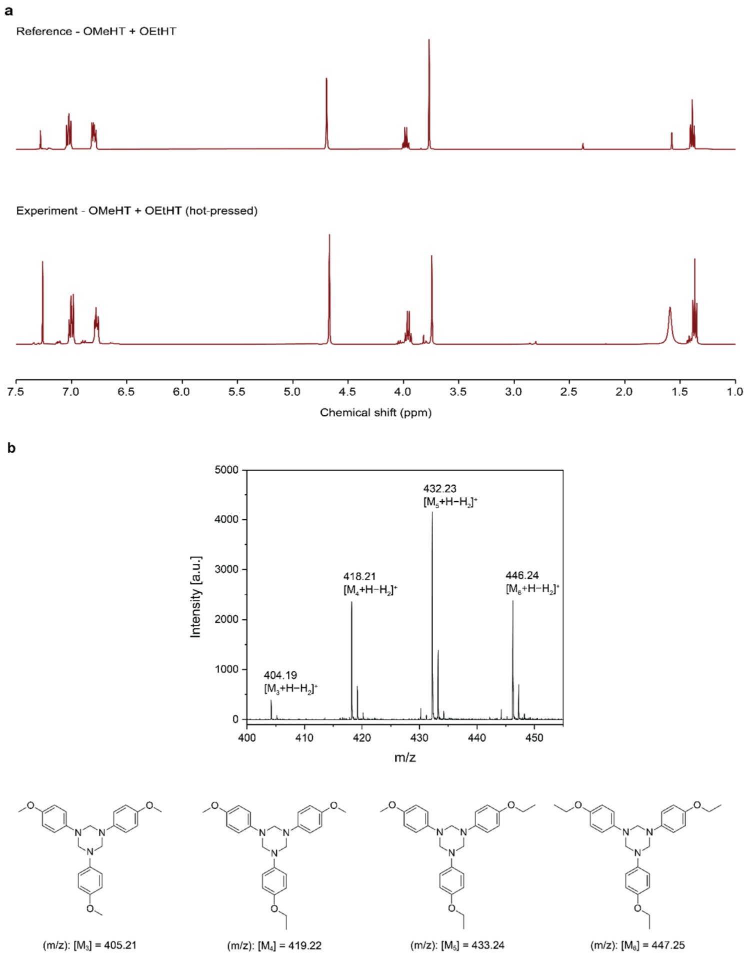

Figure S4. Hot-pressing experiment demonstrating the bond exchange reaction of two different HT (OMeHT and OEtHT) under heat and pressure. a) ^{1}H NMR spectra (400 MHz, 25 °C, $CDCl_3$) of equivalent molar ratio of OMeHT and OEtHT mixture at room temperature (top) and equivalent molar ratio OMeHT and OEtHT after hot pressed at 180 °C with 12 MPa for 0.5 h followed by 40 MPa for another 0.5 h (bottom). c) MALDI-TOF spectra of the product prepared from the reaction between OMeHT and OEtHT. OMeHT and OEtHT were mixed and hot-pressed at 180 °C with 12 MPa pressure for 0.5 h followed by 180 °C with 40 MPa pressure for 0.5 h. The identified structures are listed below the graph with their exact m/z values.

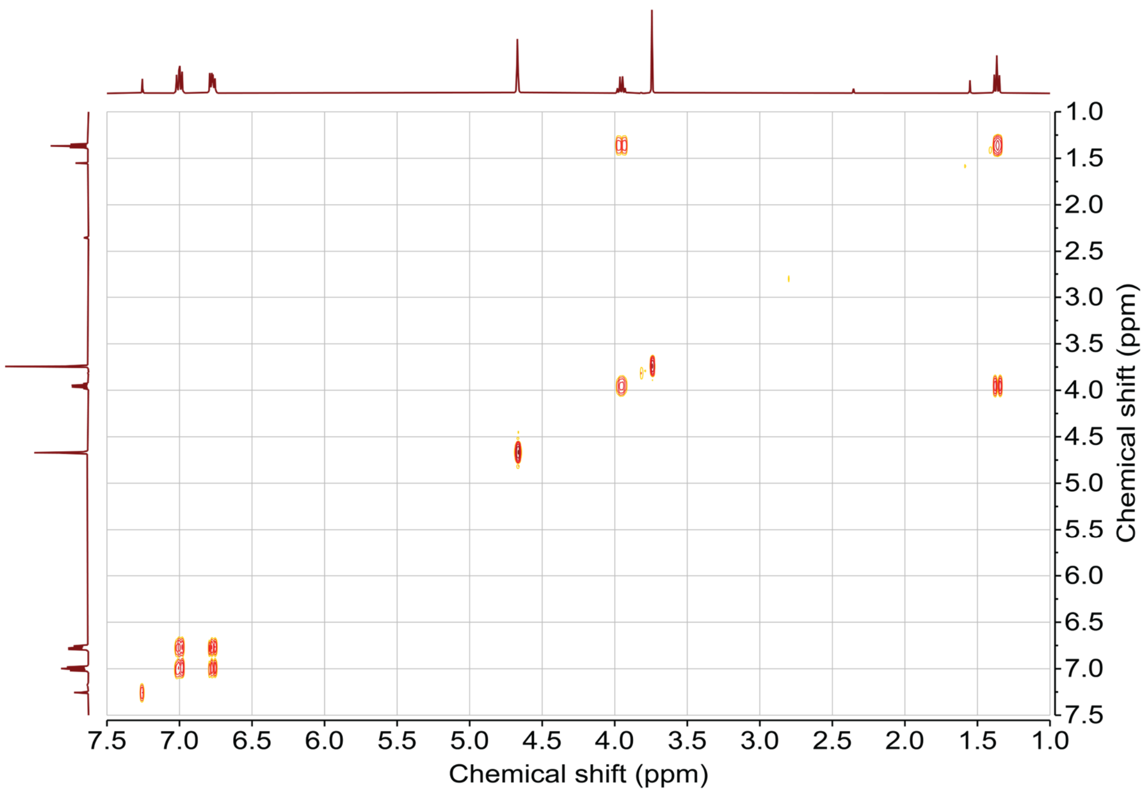

Figure S5. ^{1}H COSY NMR spectra (400 MHz, 25 °C, $CDCl_3$) of equivalent molar ratio OMeHT and OEtHT after hot-pressing experiments.

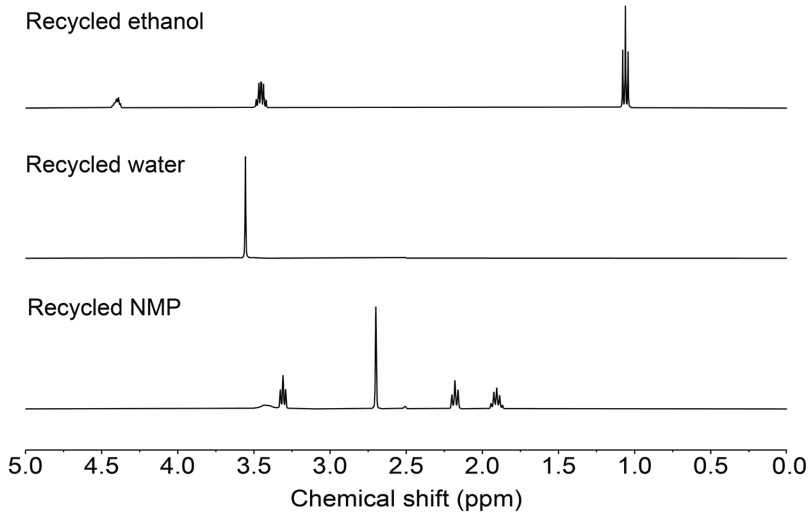

Figure S6. ^{1}H NMR spectra of distilled ethanol, distilled water, and recycled NMP (400 MHz, 25 °C, DMSO-d_6).

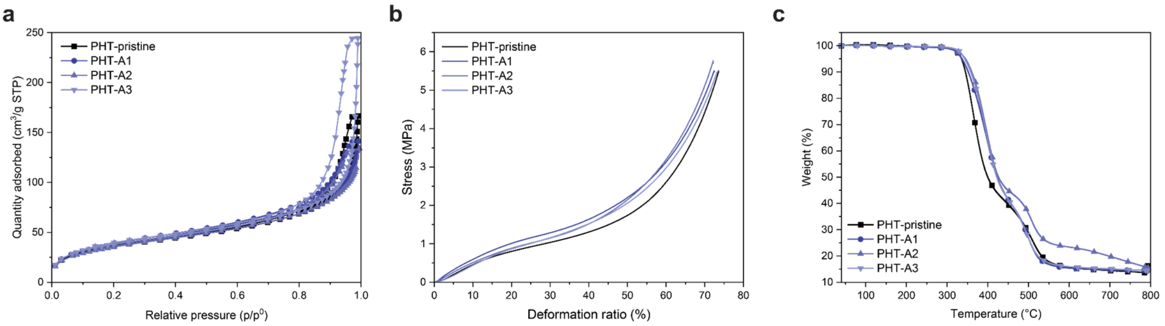

Figure S7. a) N_2 adsorption and desorption isotherms of PHT-pristine and PHT-As at 77 K. b) Stress deformation curves of PHT-pristine, PHT-A1, PHT-A2, and PHT-A3. c) TGA curves of PHT-pristine, PHT-A1, PHT-A2, and PHT-A3 ranging from 40 to 793 °C with ramp rate of 10 °C/min.

a

Name	PHT-pristine [g]	BAPP [g]	NMP [g]	Equivalent ratio of BAPP addition and BAPP in PHT	Solution mixture appearance after sonication
PHT-i	0.105	0.050	0.9	0.50	No full dissolution after 4 h sonication
PHT-ii	0.105	0.075	0.9	0.75	No full dissolution after 4 h sonication
PHT-iii	0.105	0.100	0.9	1.00	No full dissolution after 4 h sonication
PHT-iv	0.105	0.150	0.9	1.50	Full dissolution after 4 h sonication
PHT-v	0.105	0.200	0.9	2.00	Full dissolution after 4 h sonication

b

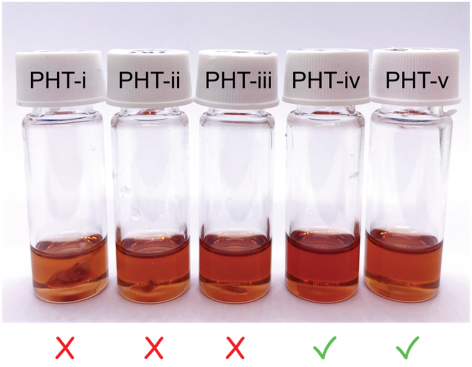

Figure S8. Investigating the minimal amount of BAPP required to partially depolymerize and thus fully dissolve PHT-pristine in NMP. a) List of ingredients used for this experiment and b) photographs showing the incomplete (PHT-i–iii) and complete (PHT-iv and PHT-v) dissolution of PHT-pristine after sonication at 65 °C.

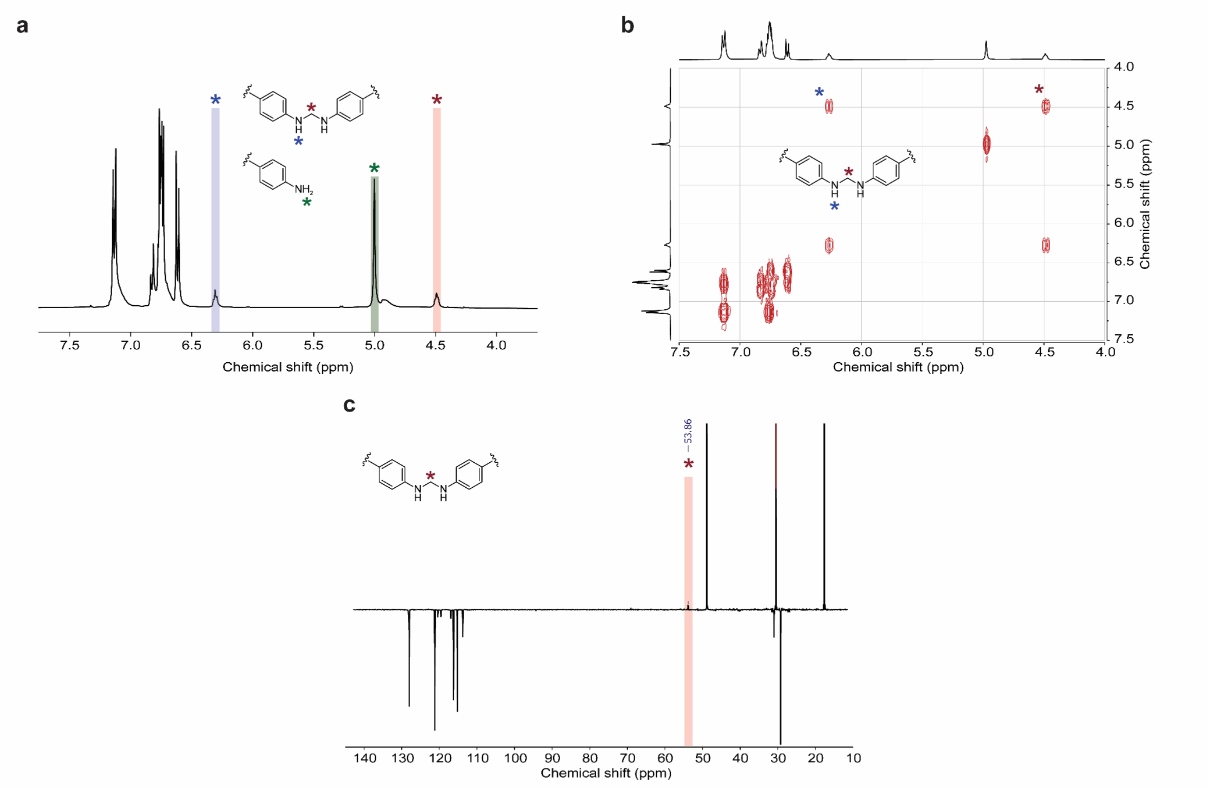

Figure S9. NMR spectroscopy experiment demonstrating the partial depolymerization of PHT-pristine. a) ^{1}H NMR spectrum (400 MHz, 25 °C, DMSO-d_6), b) ^{1}H COSY NMR spectrum (400 MHz, 25 °C, DMSO-d_6), and c) DEPT ^{13}C NMR spectrum (100 MHz, 25 °C, DMSO-d_6) after dissolution of PHT-pristine.

a

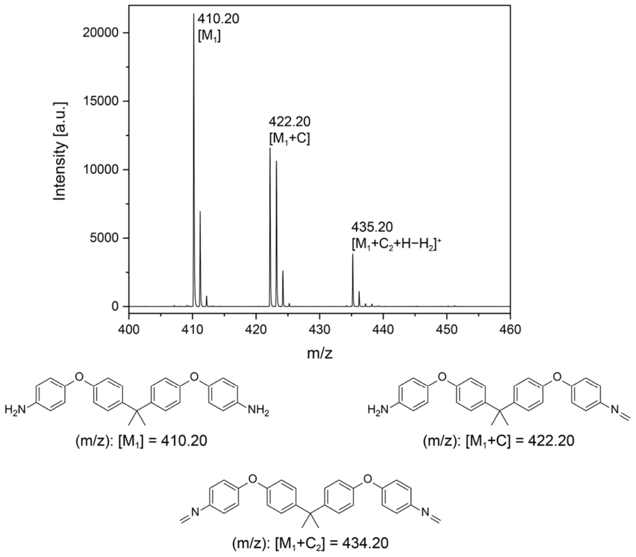

b

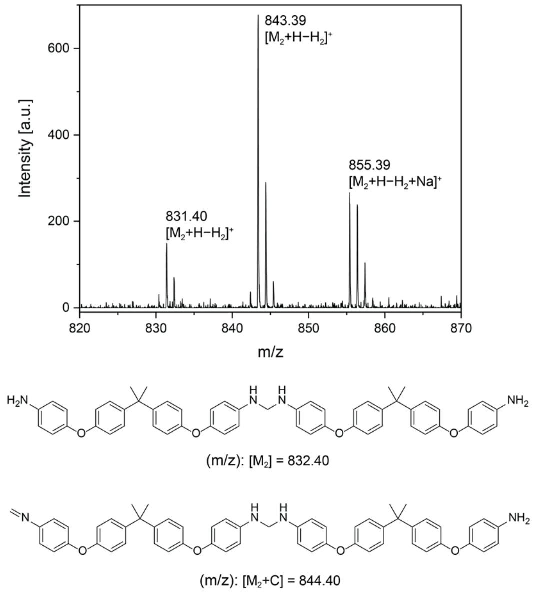

Figure S10. MALDI-TOF spectrum of the PHT-pristine dissolution in DMF. a) zoomed image of the mass spectra with m/z ranging from 400 to 460. The identified structures are listed below the graph with their exact mass. b) zoomed image of the mass spectra with m/z ranging from 820 to 870. The identified structures are listed below the graph with their exact mass.

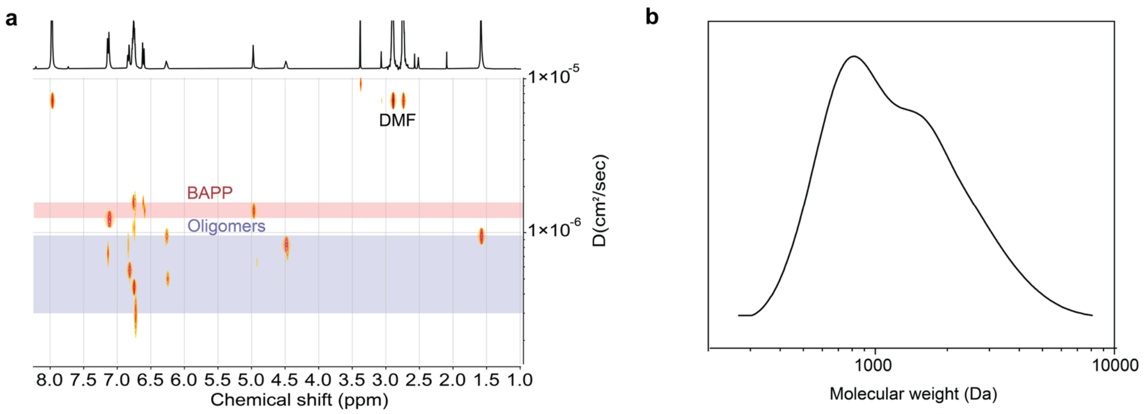

Figure S11. a) ^{1}H DOSY NMR spectrum of the solution after dissolution in DMF/DMSO-d_6. The signals of different compounds were marked in different colors (red: BAPP and blue: oligomers). b) Molecular weight distribution of PHT-pristine dissolution in DMF. The graph is obtained by GPC analysis. DMF was used as the eluent and the GPC traces were calibrated with a PMMA as standard to calculate the molecular weight.

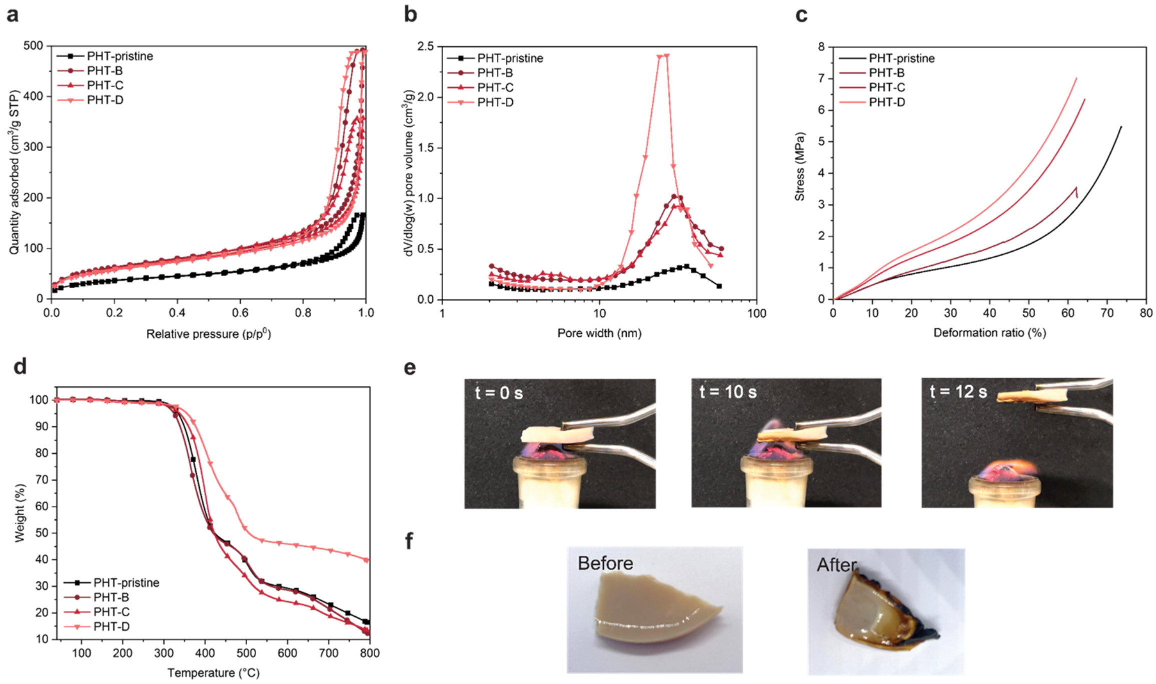

Figure S12. a) N_2 adsorption and desorption isotherms of PHT-pristine, PHT-B, PHT-C, and PHT-D at 77 K. b) BJH pore size distribution of PHT-pristine, PHT-B, PHT-C, and PHT-D. c) Stress deformation curves of PHT-pristine, PHT-B, PHT-C, and PHT-D. d) TGA curves of PHT-pristine, PHT-B, PHT-C, and PHT-D ranging from 40 to 793 °C with ramp rate of 10 °C/min. e) Photographs illustrating the burning test of PHT-D. Sample with 5 mm thickness and 10 mm width were set up horizontally and the bottom of the specimens was exposed to an ethanol burner for 10 s (**Movie S1**). f) Photographs showing the PHT-D sample before and after burning test.

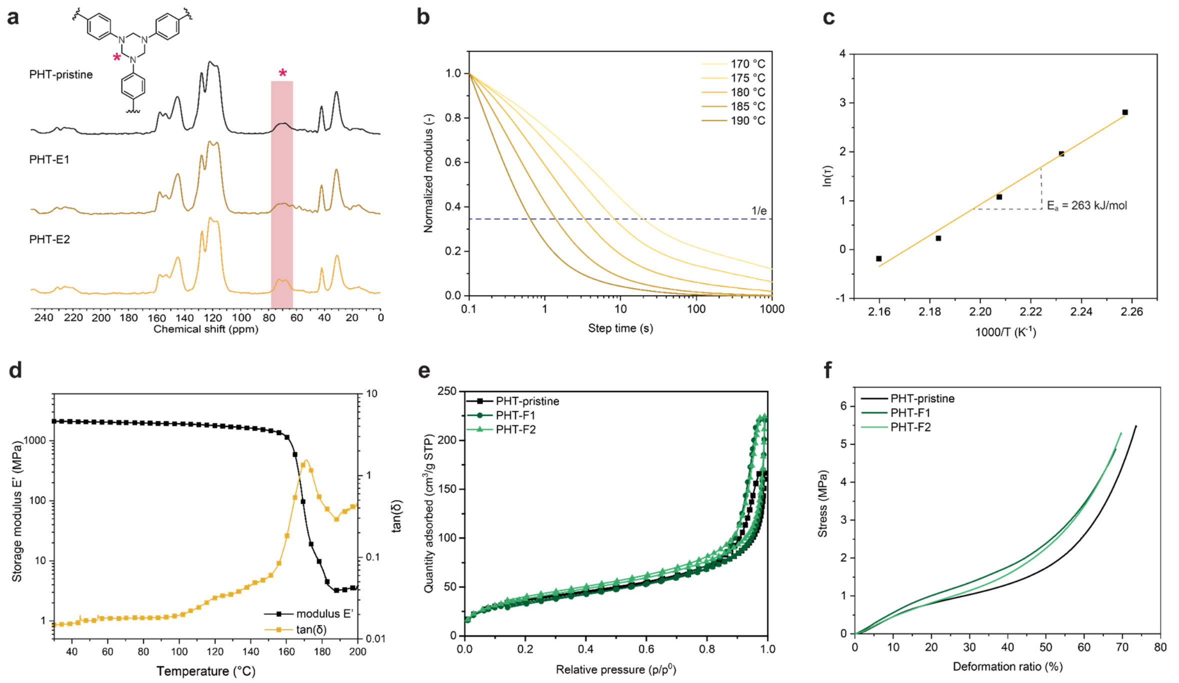

Figure S13. a) MAS ^{13}C NMR spectra of PHT-pristine and PHT-Es. b) Normalized stress-relaxation curves of PHT-E2. c) Fitted curve for PHT-E2 between 1000/T and characteristic relaxation time (ln τ*) according to the Arrhenius law. d) DMTA analysis graph of PHT-E2 showing storage moduli (MPa, black) and tan(δ) (yellow) values. e) N_2 adsorption and desorption isotherms of PHT-pristine, PHT-F1 and PHT-F2 at 77 K. f) Stress deformation curves of PHT-pristine, PHT-F1 and PHT-F2.

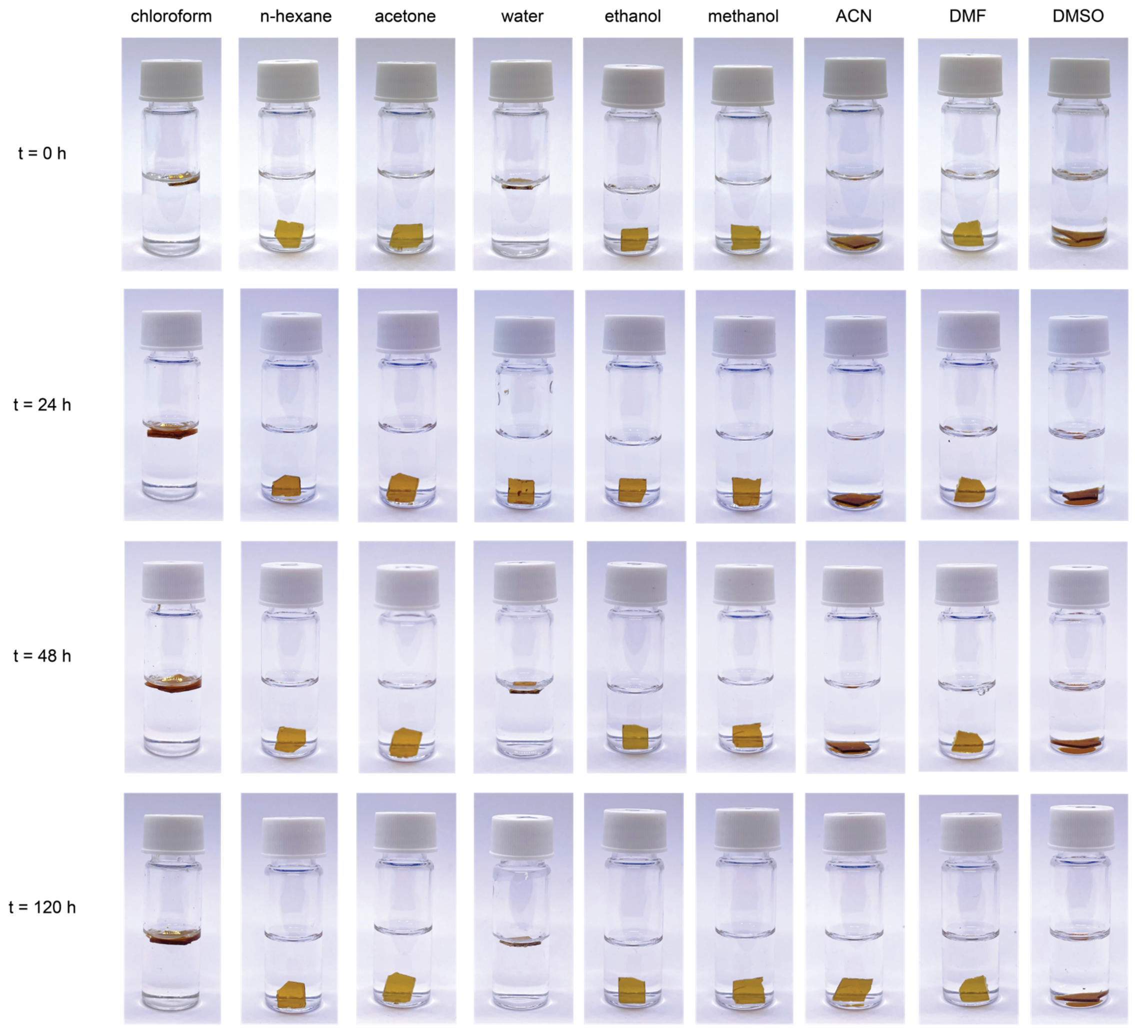

Figure S14. Photographs depicting the solvent stability of PHT-E1 in chloroform, n-hexane, acetone, water, ethanol, methanol, acetonitrile (ACN), dimethylformamide (DMF), and dimethylsulfoxide (DMSO) at t = 0 h, 24 h, 48 h, and 120 h.

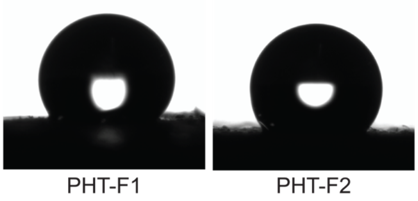

Figure S15. Water contact angle images of PHT-F1 and PHT-F2.

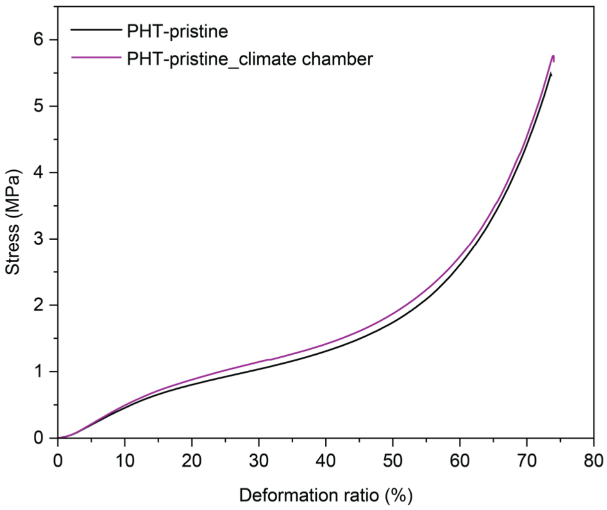

Figure S16. Stress-deformation curves of PHT-pristine after placing in a climate at 70 °C, RH 70 % for 14 days.

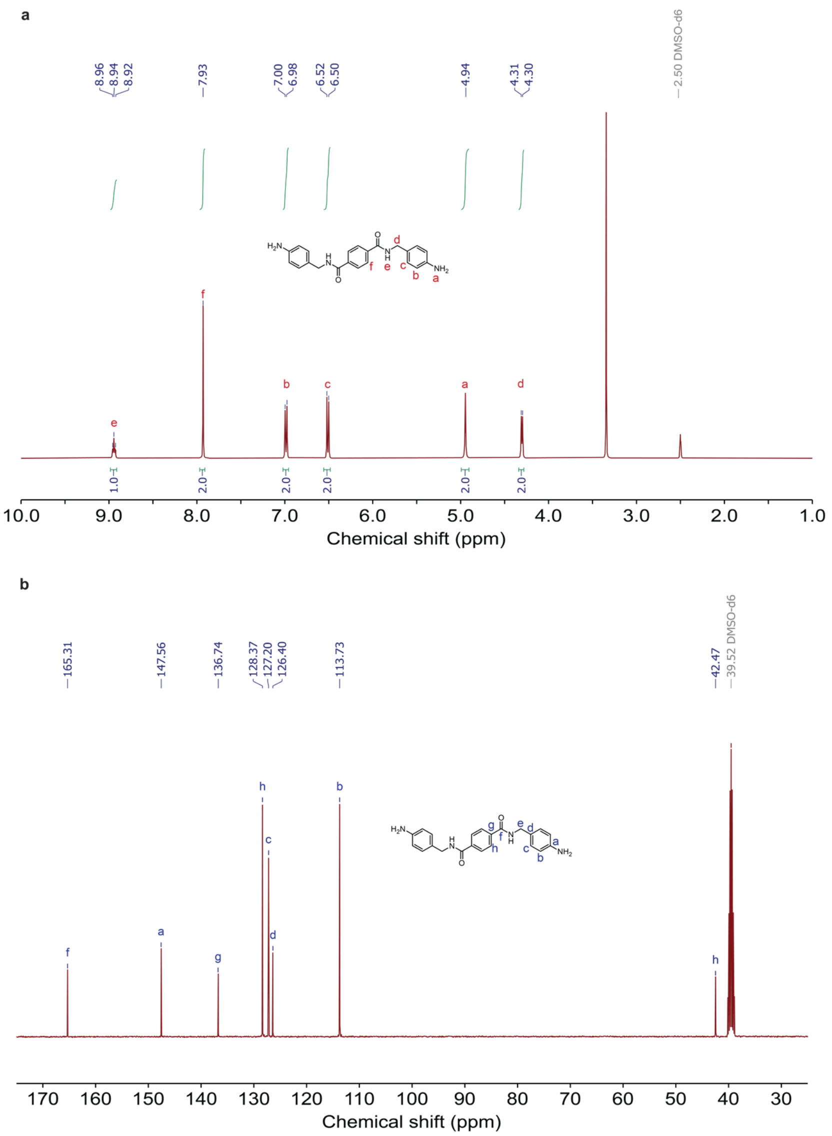

Figure S17. NMR spectra of BAPTPA in DMSO-d_6 as solvent. a) ^{1}H NMR spectra (400 MHz, 25 °C). b) ^{13}C NMR spectra (100 MHz, 25 °C).

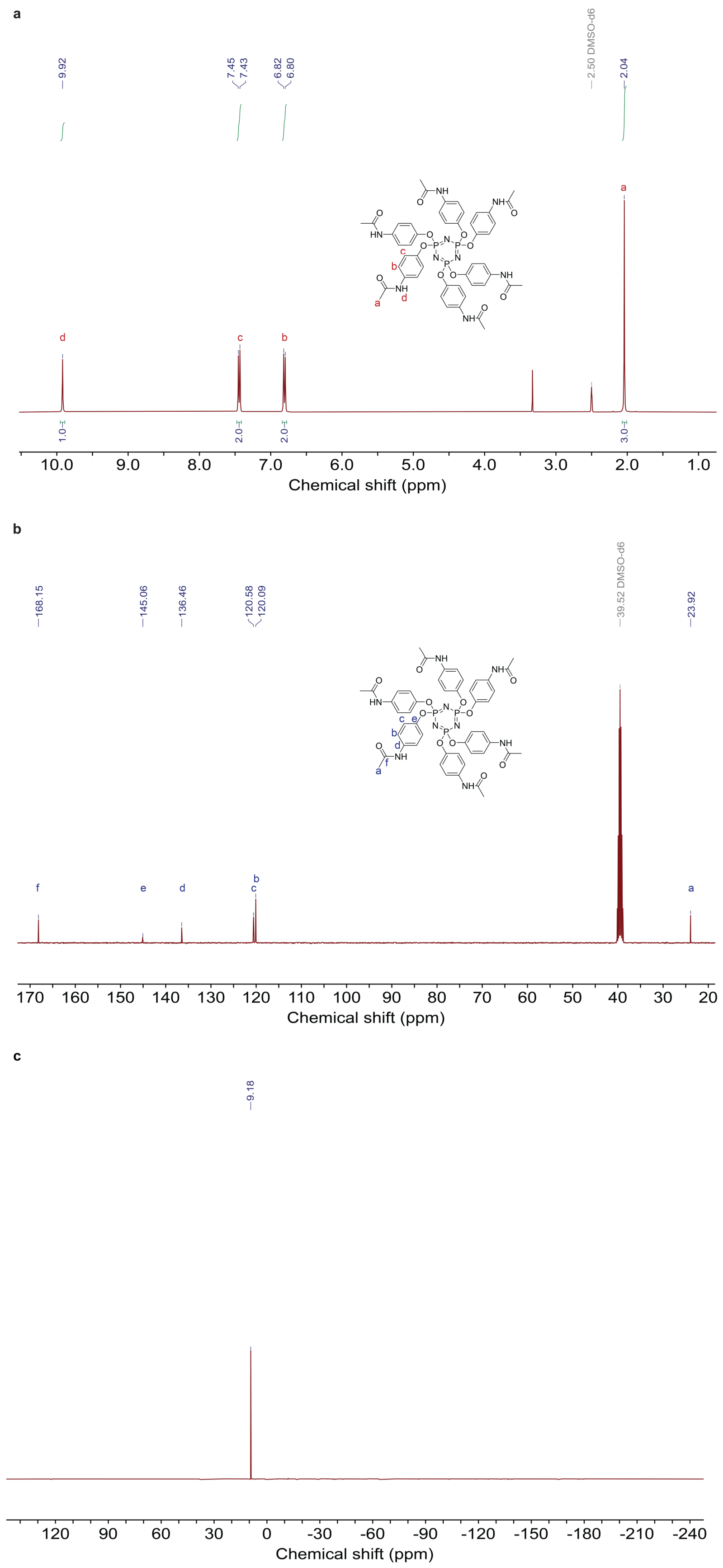

Figure S18. NMR spectra of HAAPP in DMSO-d_6 as solvent. a) ^{1}H NMR spectra (400 MHz, 25 °C). b) ^{13}C NMR spectra (100 MHz, 25 °C). c) ^{31}P NMR spectra (161.9 MHz, 25 °C).

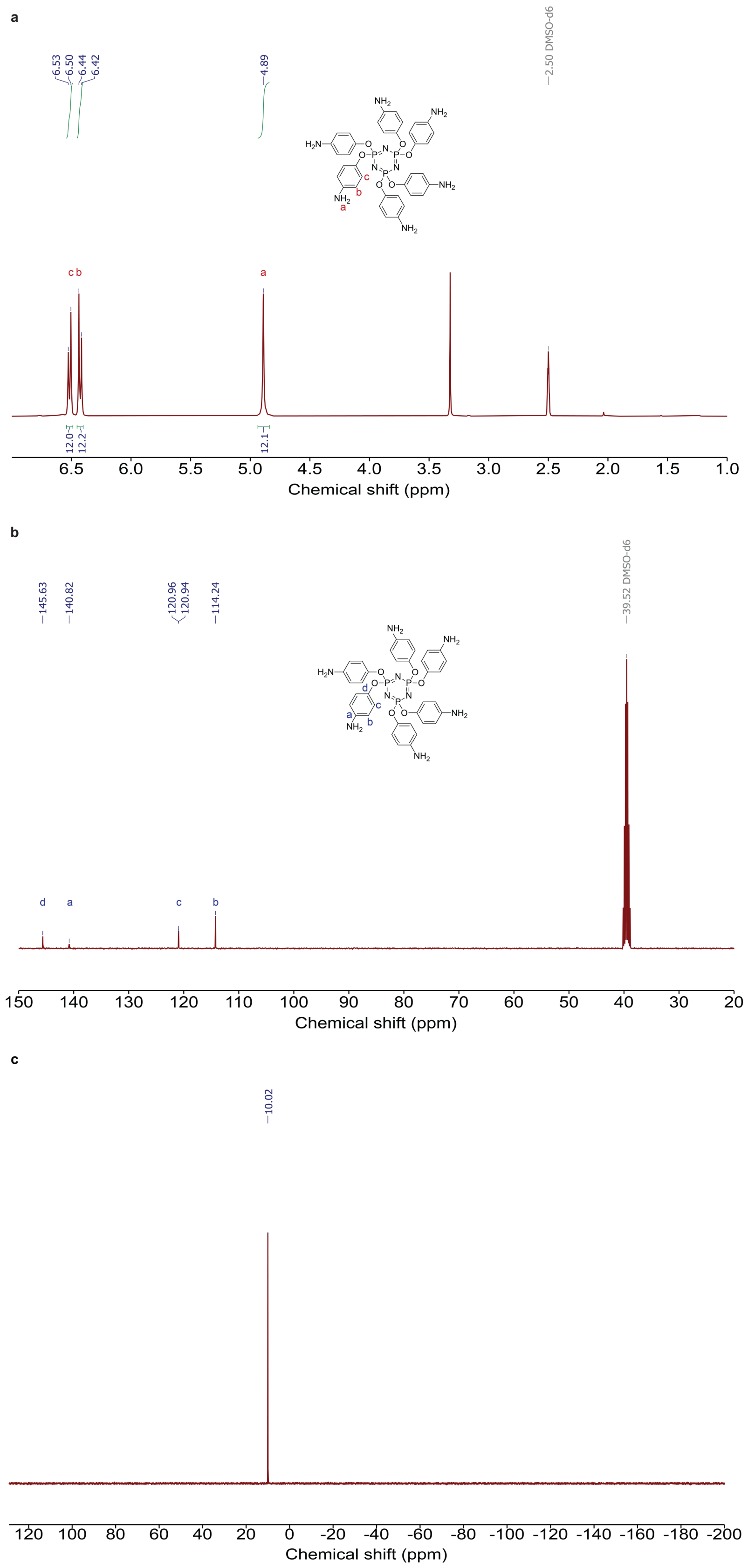

Figure S19. NMR spectra of HAPP in DMSO-*d₆* as solvent. a) ^{1}H NMR spectra (400 MHz, 25 °C). b) ^{13}C NMR spectra (100 MHz, 25 °C). c) ^{31}P NMR spectra (161.9 MHz, 25 °C).

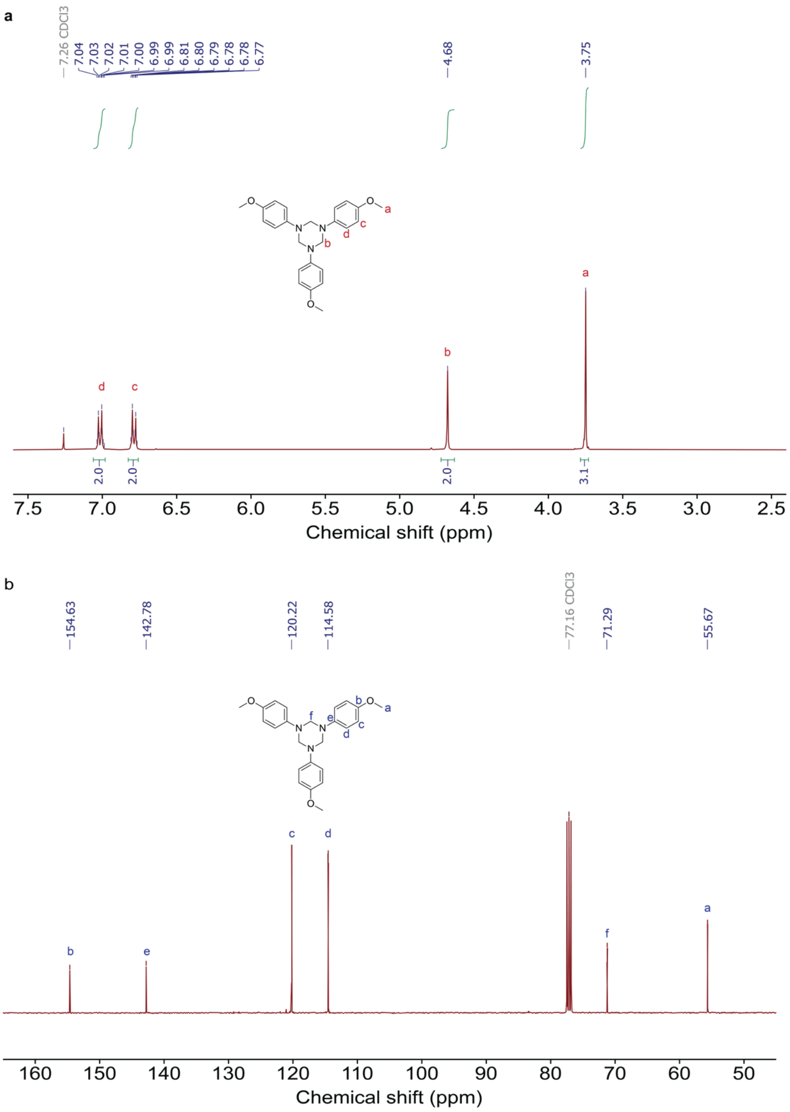

Figure S20. NMR spectra of OMeHT in $CDCl_3$ as solvent. a) ^{1}H NMR spectra (400 MHz, 25 °C). b) ^{13}C NMR spectra (100 MHz, 25 °C).

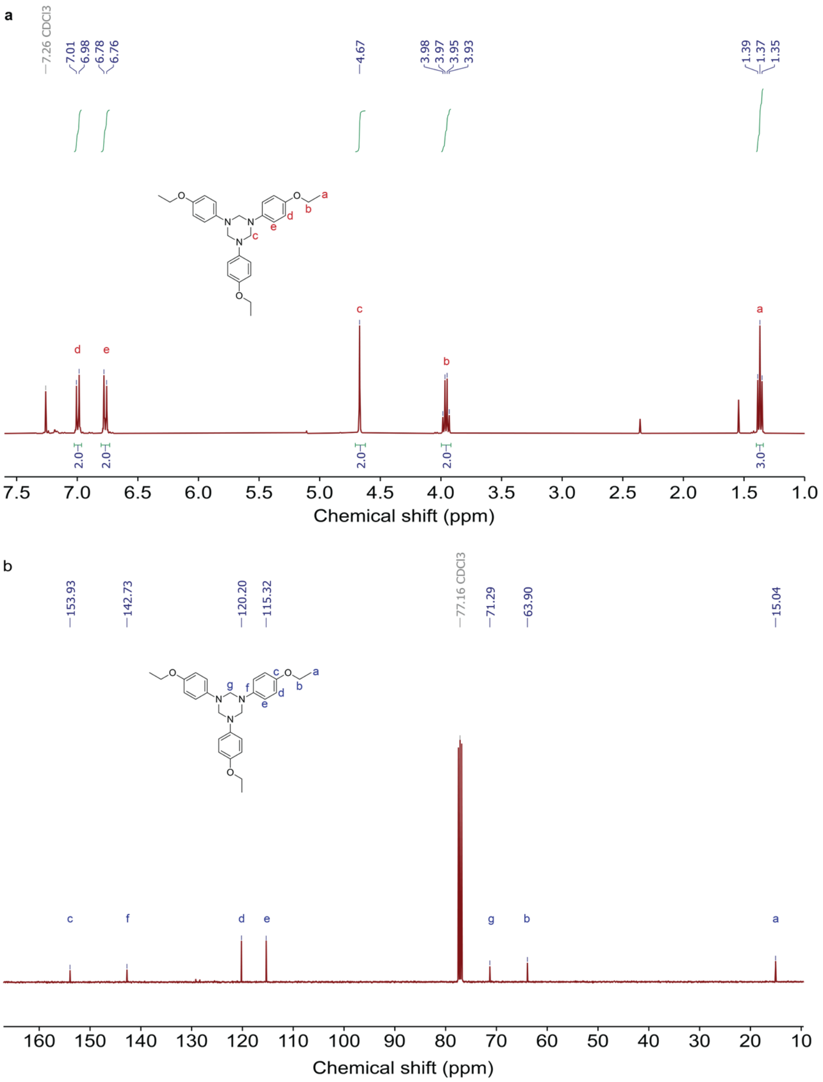

Figure S21. NMR spectra of OEtHT in $CDCl_3$ as solvent. a) ^{1}H NMR spectra (400 MHz, 25 °C). b) ^{13}C NMR spectra (100 MHz, 25 °C).

References

1. Wang, C., Eisenreich, F. & Tomović, Ž. Closed-Loop Recyclable High-Performance Polyimine Aerogels Derived from Bio-Based Resources. *Adv. Mater.* **35**, 2209003 (2023).
2. Fukushima, K. *et al.* Advanced chemical recycling of poly(ethylene terephthalate) through organocatalytic aminolysis. *Polym. Chem.* **4**, 1610–1616 (2013).
