# Supplementary material for: Advancing aerogel recyclability through polyhexahydrotriazine reactivity
Source: Nat Commun. 2025 Dec 5;17:371. doi: 10.1038/s41467-025-67059-y (PMC12796227; doi:10.1038/s41467-025-67059-y)
Supplement: Supplementary file 2 — Description of additional supplementary files [file 41467_2025_67059_MOESM2_ESM.pdf]

Description of Additional Supplementary Files

Supplementary Movie 1 :

Recording of the burning test of the PHT-D sample using an ethanol burner for 10 seconds.
